# Crystal structure, Hirshfeld surface analysis, and DFT and mol­ecular docking studies of 6-cyanona­phthalen-2-yl 4-(benz­yloxy)benzoate

**DOI:** 10.1107/S2056989024009964

**Published:** 2024-10-22

**Authors:** Mahadevaiah Harish Kumar, Shivakumar Santhosh Kumar, Hirehalli Chikkegowda Devarajegowda, Hosapalya Thimmaiah Srinivasa, Bandrehalli Siddagangaiah Palakshamurthy

**Affiliations:** a Department of Physics Yuvaraja's College University of Mysore,Mysore 570005 Karnataka India; b Department of PG Studies and Research in Physics Albert Einstein Block UCS Tumkur University, Tumkur Karnataka-572103 India; c Raman Research Institute, C V Raman Avenue Sadashivanagar Bangalore Karnataka India; Vienna University of Technology, Austria

**Keywords:** crystal structure, Hirshfeld surface, DFT, 4-(benz­yloxy)benzoate, cyanona­pthalene and mol­ecular docking, inter­molecular inter­actions

## Abstract

In the title compound, C_25_H_17_NO_3_, the torsion angle associated with the phenyl benzoate group is −173.7 (2)° and that for the benz­yloxy group is −174.8 (2)° establishing an *anti*-type conformation. The dihedral angles between the ten-membered cyanona­phthalene ring and the aromatic ring of the phenyl benzoate and the benz­yloxy fragments are 40.70 (10) and 87.51 (11)°, respectively, whereas the dihedral angle between the aromatic phenyl benzoate and the benz­yloxy fragments is 72.30 (13)°. In the crystal, the mol­ecules are linked by weak C—H⋯O inter­actions forming *S*(4) chains propagating parallel to [010]. The packing is consolidated by three C—H⋯π inter­actions and two π–π stacking inter­actions between the aromatic rings of naphthalene and phenyl benzoate with centroid-to-centroid distances of 3.9698 (15) and 3.8568 (15) Å, respectively. Inter­molecular inter­actions were qu­anti­fied using Hirshfeld surface analysis. The mol­ecular structure was further optimized by density functional theory (DFT) at the B3LYP/6–311+ G(d,p) level, revealing that the energy gap between HOMO and LUMO is 3.17 eV. Mol­ecular docking studies were carried out for the title compound as a ligand and SARS-Covid-2(PDB ID:7QF0) protein as a receptor giving a binding affinity of −9.5 kcal mol^−1^.

## Abstract 2

The crystal structure of the title compound is consolidated by C—H⋯O, C—H⋯π and π–π stacking inter­actions. Hirshfeld surface analysis indicates that dispersion energy makes a dominate contribution to the isosurface.

## 1. Chemical context

Naphthalene derivatives play a vital role in drug design because they have shown to exhibit anti-microbial (El *et al.*, 2018 ▸), anti-cancer (Valente *et al.*, 2014 ▸), anti-viral (Perrone *et al.*, 2015 ▸), anti­convulsant (Özdemir *et al.*, 2019 ▸), anti-tubercular (Das *et al.*, 2007 ▸), anti-inflammatory (Boyle *et al.*, 1982 ▸) and anti-bacterial activities (Ashraf *et al.*, 2019 ▸). These properties are attributed to the naphthalene moiety because it is able to disrupt cell membranes, inter­fere with cell wall synthesis and inhibit enzyme activity. In this context, cyanona­phthalene derivatives have been explored for their possible anti-cancer properties (Hekal *et al.*, 2024 ▸). These compounds can cause programmed cell death in cancer cells, which can help slow down tumour growth. They have also been shown to have anti­fungal activity (Prakash *et al.*, 2015 ▸) and to operate as potential inhibitors for the treatment of congestive heart failure and cardiac fibrosis (Voets *et al.*, 2005 ▸), or against plant pathogenic fungi (Jin *et al.*, 2024 ▸). Biological activities usually vary depending on the mol­ecular structure of the compound, its substitution pattern, and strains used. In this regard, benz­yloxy derivatives demonstrate anti-malarial, anti-platelet, and anti-bacterial activities (Mohebi *et al.*, 2022 ▸; de Candia *et al.*, 2015 ▸; Kaushik *et al.*, 2018 ▸), while pyrimidinyl­phenyl­amine-substituted benzo­yloxy derivatives are most potent in inhibiting HIV-1 (Rai *et al.*, 2023 ▸).
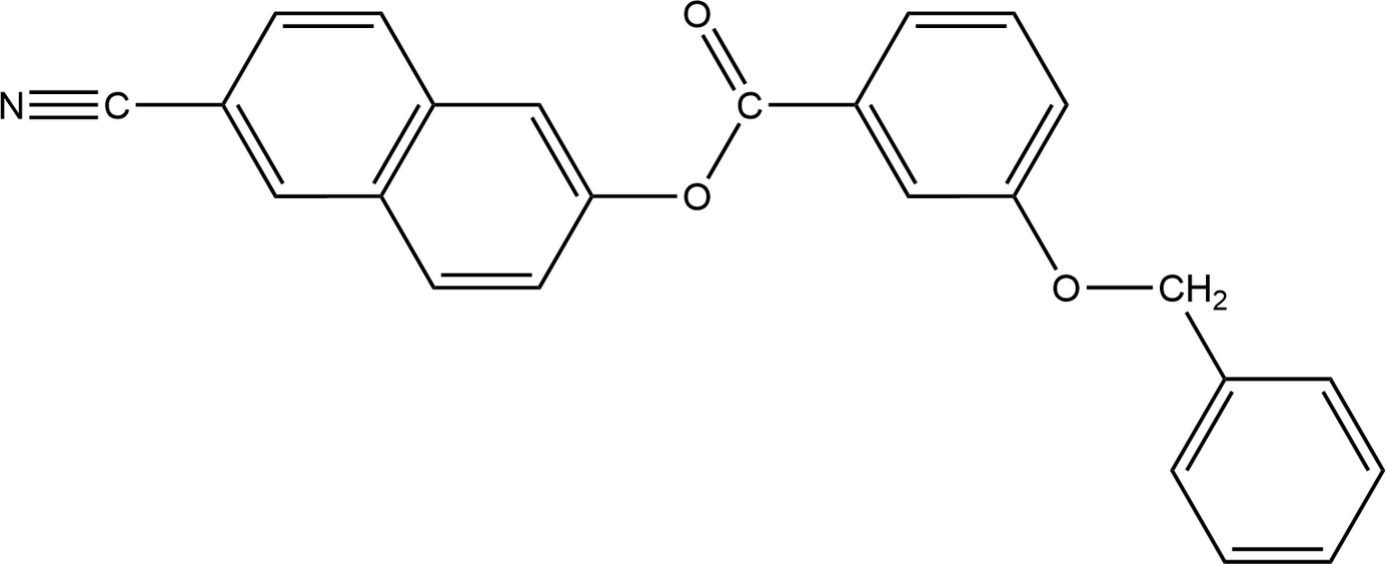


In order to explore cyanona­phthalene and (benz­yloxy)benzoate groups, we have adopted these moieties for the formation of organic liquid-crystal materials (Srinivasa *et al.*, 2020 ▸). However, the toxicity of naphthalene and its potential carcinogenic properties may limit its use, and more research is needed to fully understand the mechanisms of potential action for therapeutic applications.

In the context given above, we present here the synthesis and structure elucidation of the cyanona­phthalene derivative, C_25_H_17_NO_3_, (I).

## 2. Structural commentary

**Figure 1:**
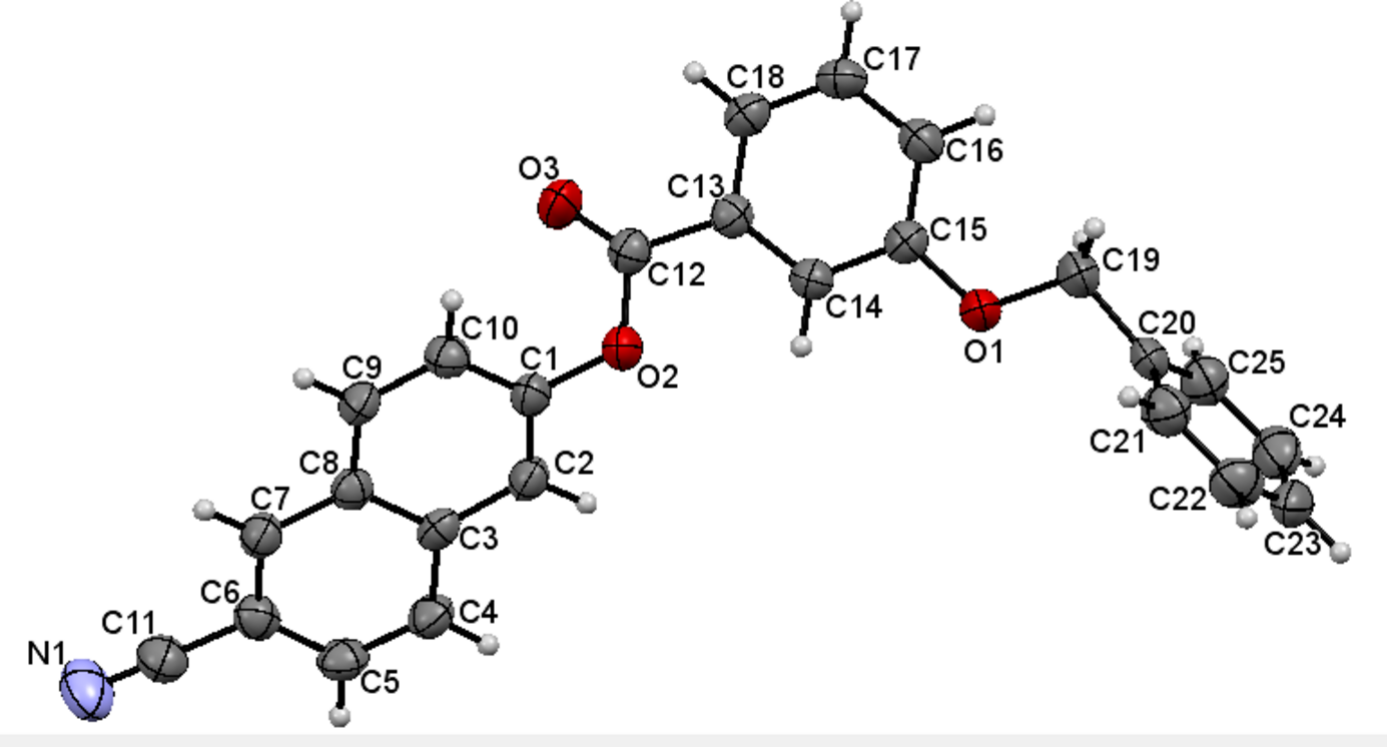
The mol­ecular structure of (I) with displacement ellipsoids drawn at the 50% probability level.

The mol­ecular structure of (I) is shown in Fig. 1 ▸. The cyanona­phthalene moiety (C1–C10, C11≡N1) is nearly planar with an r.m.s. deviation of 0.0762 Å, with a maximum deviation of −0.138 (2) for N1. The aromatic rings of the naphthalene system are inclined towards each other with a dihedral angle of 3.82 (12)^o^. The torsion angles at the phenyl benzoate group (C1–O2–C12–C13) and the benz­yloxy fragment (C15–O1–C19–C20) are −173.7 (2) and −174.8 (2)°, respectively, establishing an *anti*-type conformation. Otherwise, bond lengths and angles can be regarded as normal. The dihedral angle between the ten membered cyanona­phthalene ring (C1–C10) and the aromatic ring of the phenyl benzoate moiety (C13–C18) is 40.70 (10)° and between that of the benz­yloxy fragments (C20–C25) is 87.51 (11)°. The dihedral angle between the phenyl rings of the phenyl benzoate and the benz­yloxy systems is 72.30 (13)°.

## 3. Supra­molecular features

**Figure 2:**
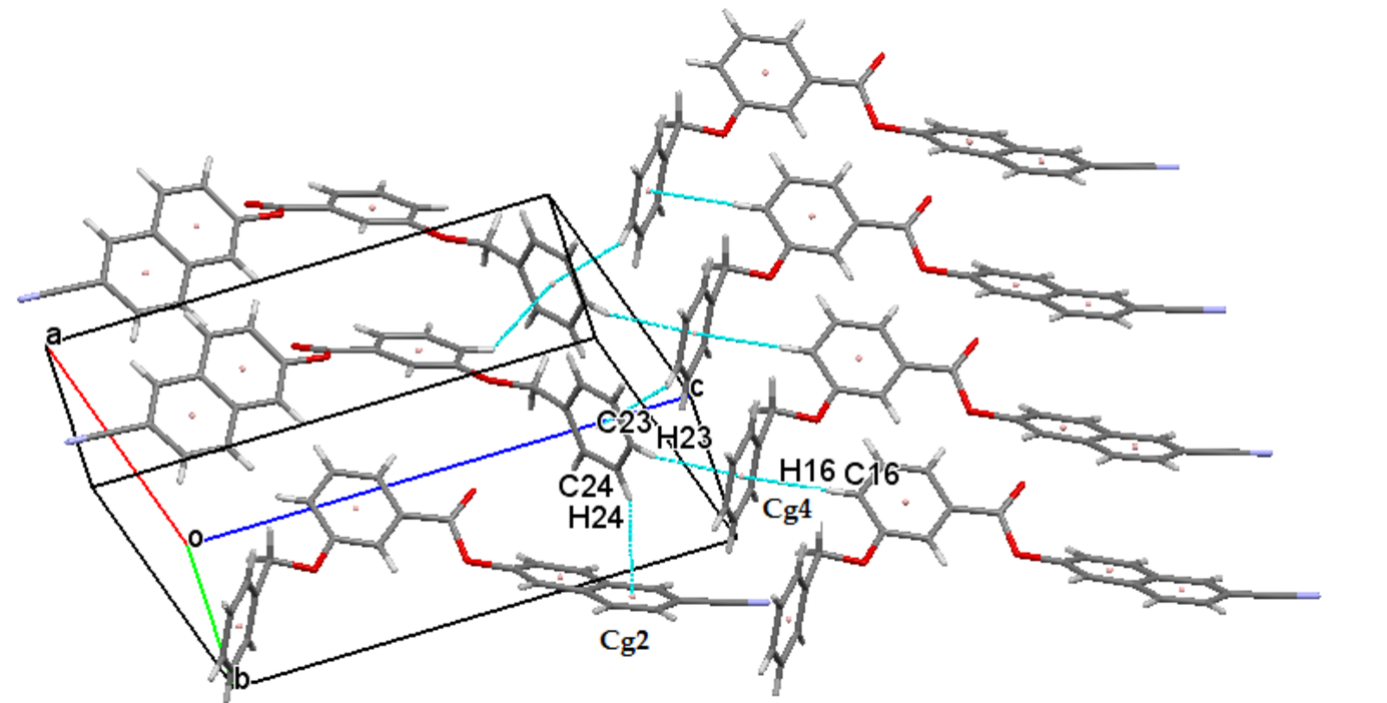
The mol­ecular packing of (I) with C—H ⋯π inter­actions depicted by dashed lines.

**Figure 3:**
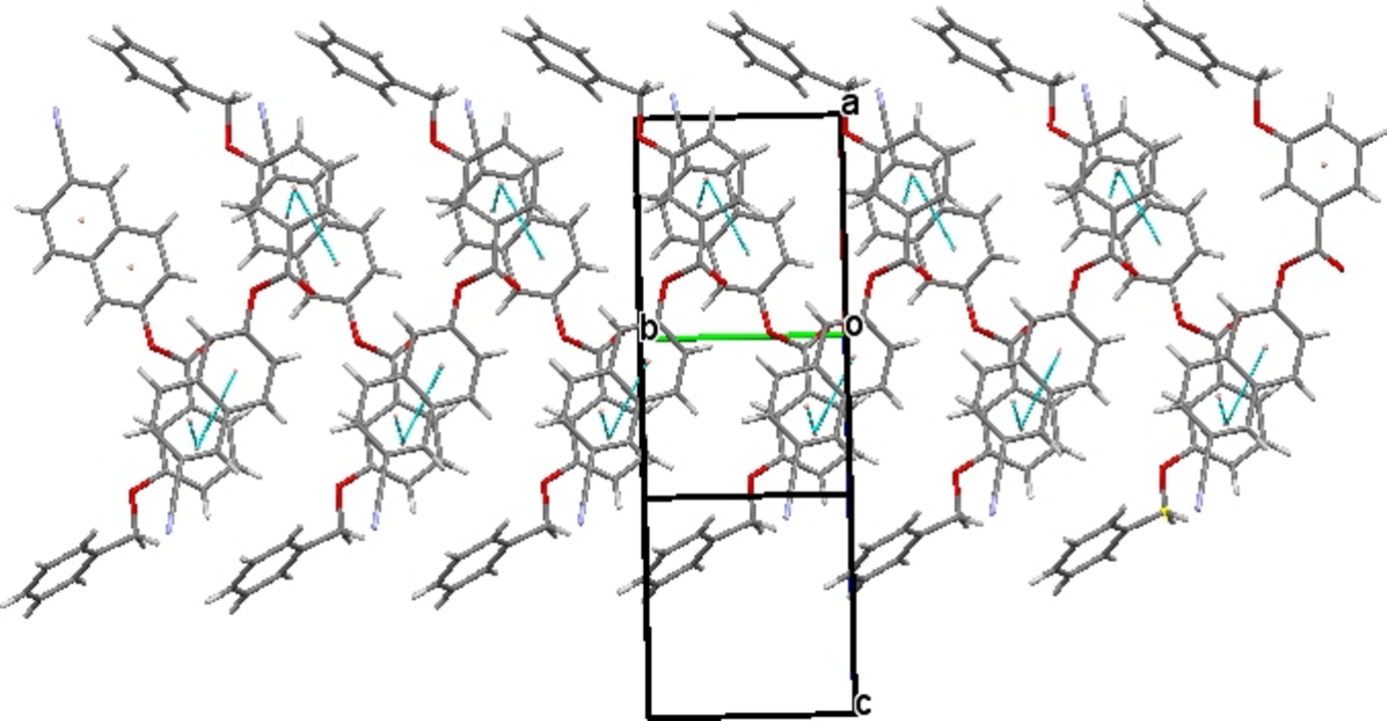
The mol­ecular packing of (I) with π–π inter­actions depicted by pale-green dashed lines.

**Figure 4:**
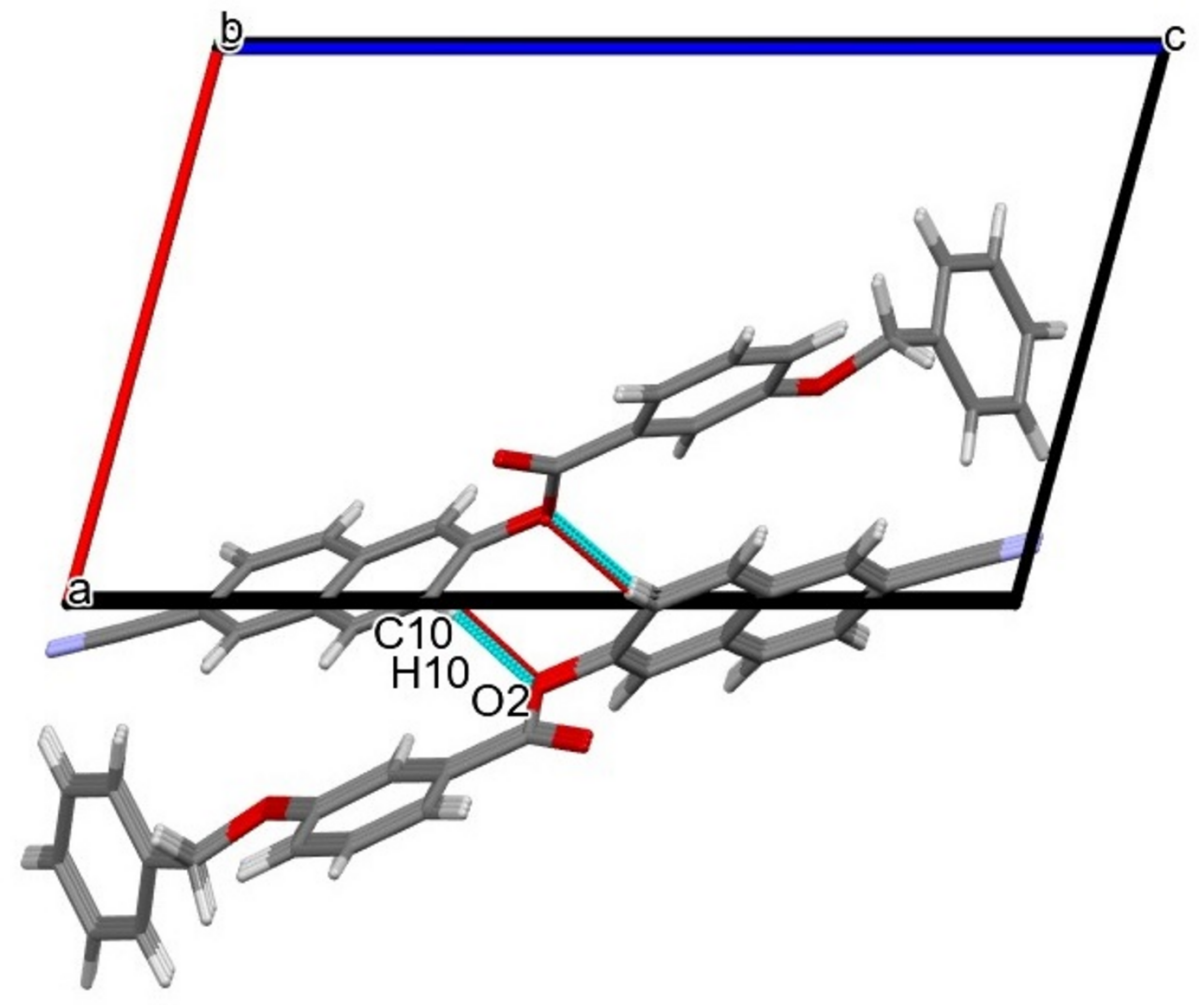
C—H⋯O inter­action in (I) forming an *S*(4) chain running parallel to [010]; symmetry code as in Table 2 ▸.

The crystal packing of (I) includes C—H⋯π inter­actions between aromatic H atoms and phenyl rings, as detailed for the inter­actions C16—H16⋯π, C23—H23⋯π and C24—H24⋯π in Table 1 ▸ and shown in Fig. 2 ▸. There are also slipped π–π inter­actions in the crystal between the two aromatic rings of the naphthalene ring system [*Cg*1 and *Cg*2 are the centroids of the C1–C3/C8–C10 and = C3–C8 rings, respectively] and phenyl benzoate ring [*Cg*3 is the centroid of the C13–C18 ring], with centroid-to-centroid distances for *Cg*1⋯*Cg*3 and *Cg*2⋯*Cg*3 of 3.9699 (15) Å (slippage 1.893 Å) and 3.8569 (10) Å (slippage 1.731 Å), respectively, as shown in Fig. 3 ▸. In addition, a weak C10—H10⋯O2 inter­action (Table 1 ▸) forming a chain parallel to [010] with an *S*(4) motif (Bernstein *et al.*, 1995 ▸) is an integral part of the crystal packing (Fig. 4 ▸).

## 4. Hirshfeld surface analysis

**Figure 5:**
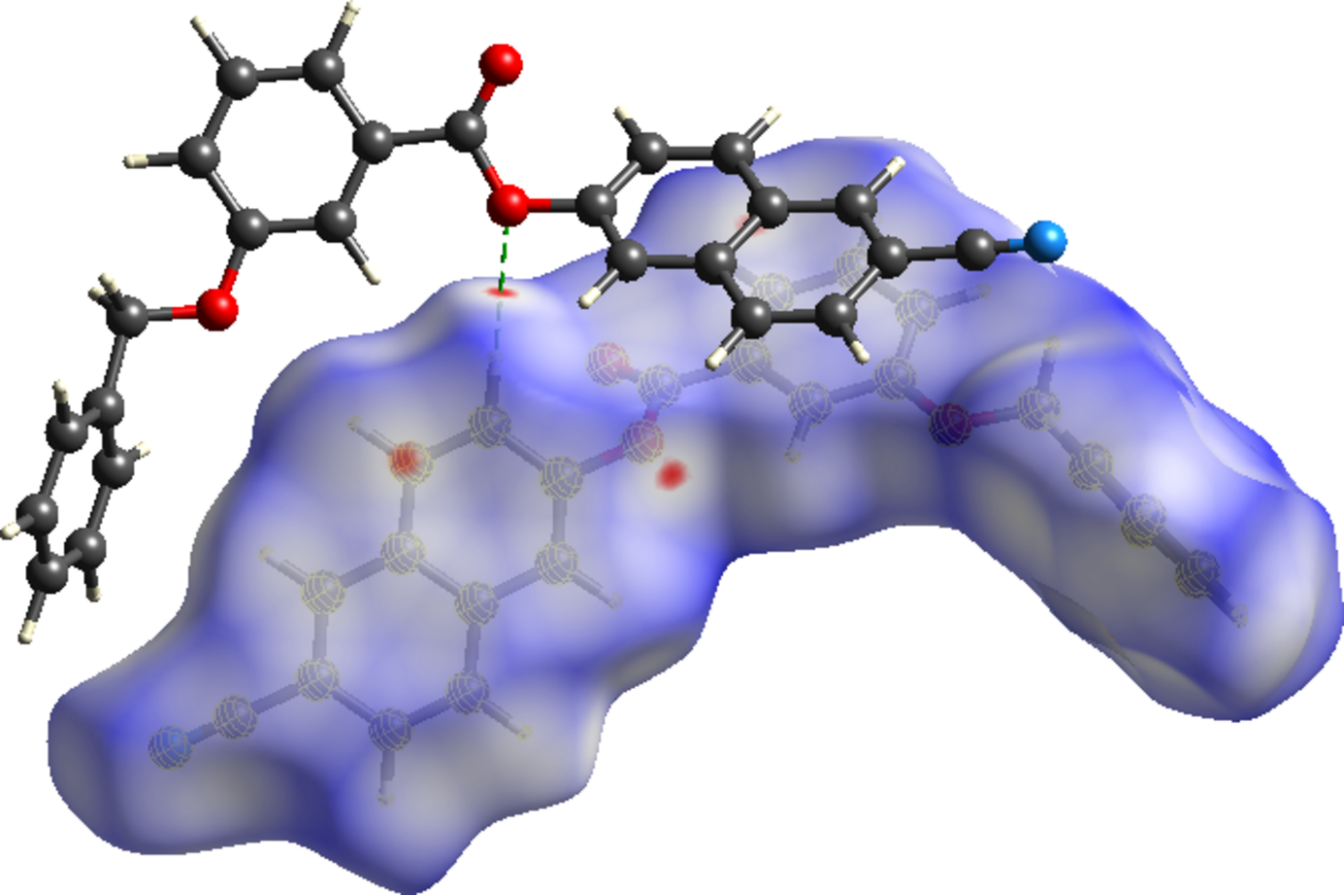
Hirshfeld surface of (I) plotted over *d*_norm_; the dashed lines indicate the C—H⋯O inter­actions.

**Figure 6:**
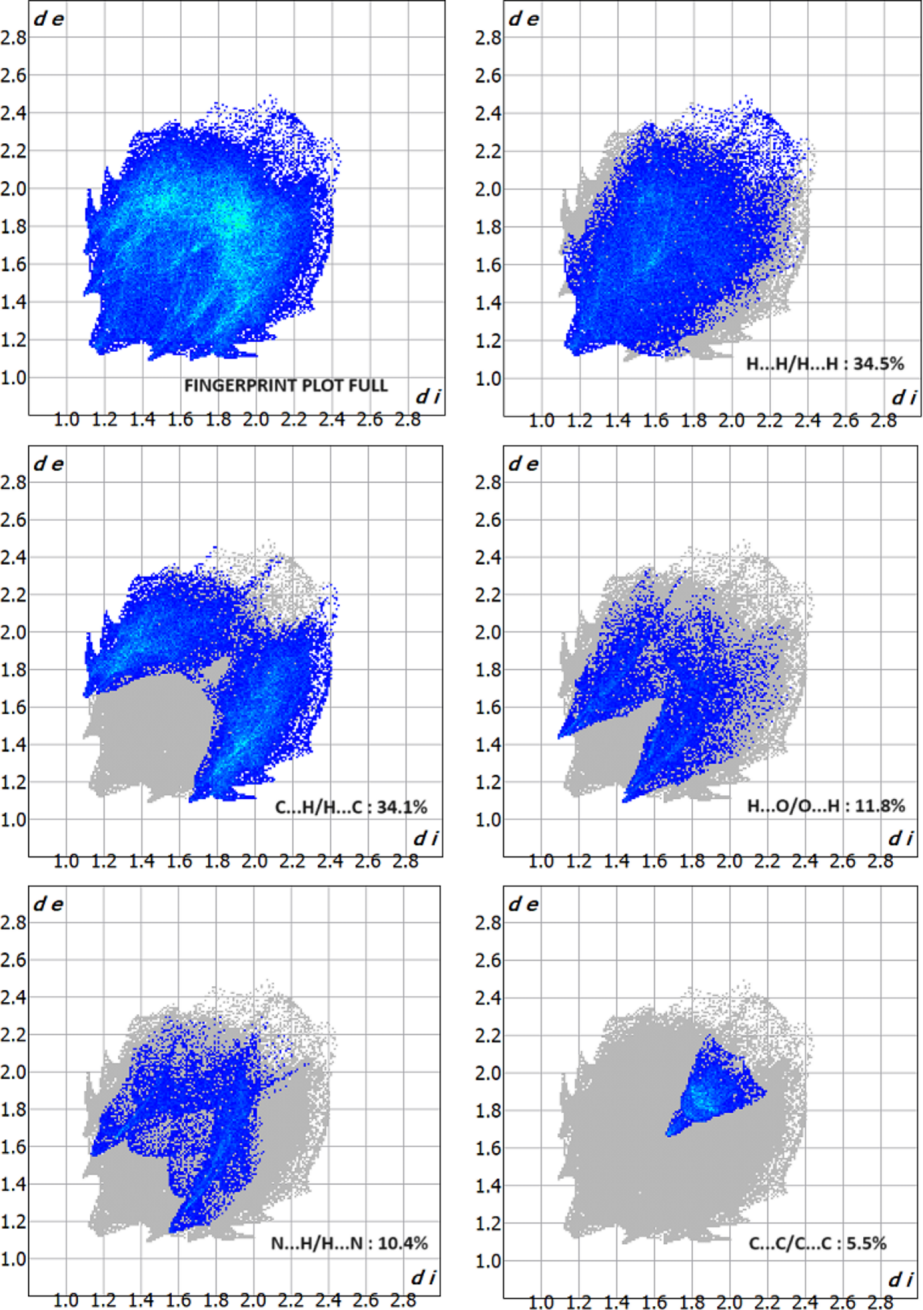
Two-dimensional fingerprint plots for the title compound, showing all inter­actions, and delineated into H⋯H, C⋯H/H⋯C, H⋯O/O⋯H, N⋯H/H⋯N, and C⋯C inter­actions.

Hirshfeld surface analysis (Hirshfeld, 1977 ▸; Spackman & Jayatilaka, 2009 ▸) was used to visualize and qu­antify inter­molecular inter­actions using *CrystalExplorer* (Spackman *et al.*, 2021 ▸). Fig. 5 ▸ illustrates the Hirshfeld surface mapped over *d*_norm_, where the colour code denotes inter­molecular inter­actions on the Hirshfeld surface: the contacts with distances equal to the sum of the van der Waals radii are indicated in white, while those with shorter and longer distances are represented in red and blue, respectively. For (I), the C10—H10⋯O2 inter­action is responsible for the red regions. The two-dimensional fingerprint plots indicate that the major contributions to the crystal packing of (I) are from H⋯H (34.5% contribution), C⋯H/H⋯C (34.1%), O⋯H/H⋯O (11.8%), N⋯H/H⋯N (10.4%) and C⋯C (5.5%) contacts, as shown in Fig. 6 ▸.

## 5. Density functional theory (DFT) studies

**Figure 7:**
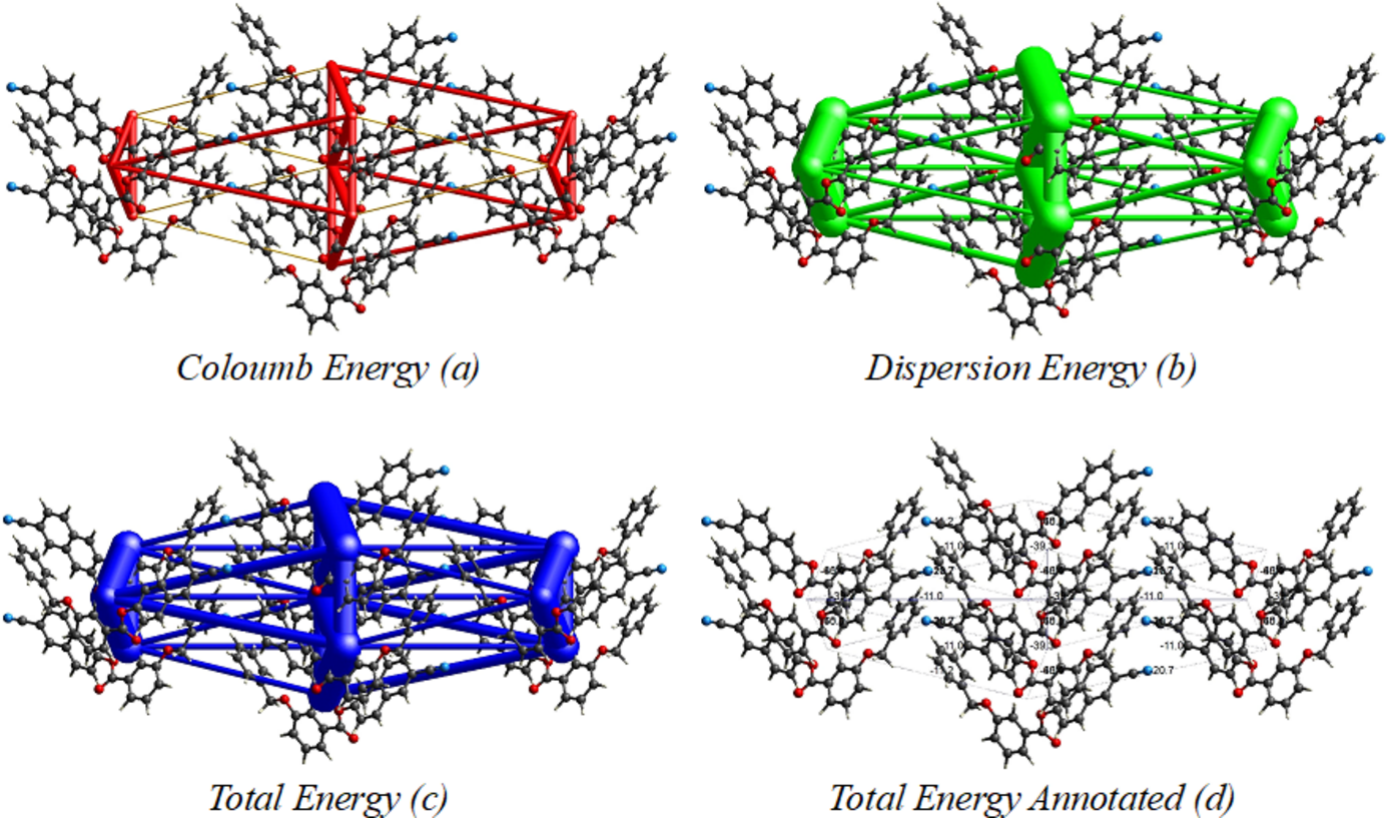
Energy frameworks calculated for the title compound, viewed along the *a* axis direction, showing (*a*) Coulomb potential force, (*b*) dispersion force and (*c*, *d*) total energy diagrams. The cylindrical radii are proportional to the relative strength of the corresponding energies; they were adjusted to a cutoff value of 5 kJ mol^−1^.

Energies were computed using the basis set B3LYP\631-G(d,p). The net inter­action energies are *E*_ele_ = 55.2 kJ mol^−1^, *E*_pol_ = 17.1 kJ mol^−1^, *E*_dis_ = 218.8 kJ mol^−1^, *E*_rep_ = 105.8 kJ mol^−1^ and the total inter­action energy *E*_tot_ = 189.9 kJ mol^−1^. The topology of energy frameworks for inter­action energies are shown in Fig. 7 ▸.

**Figure 8:**
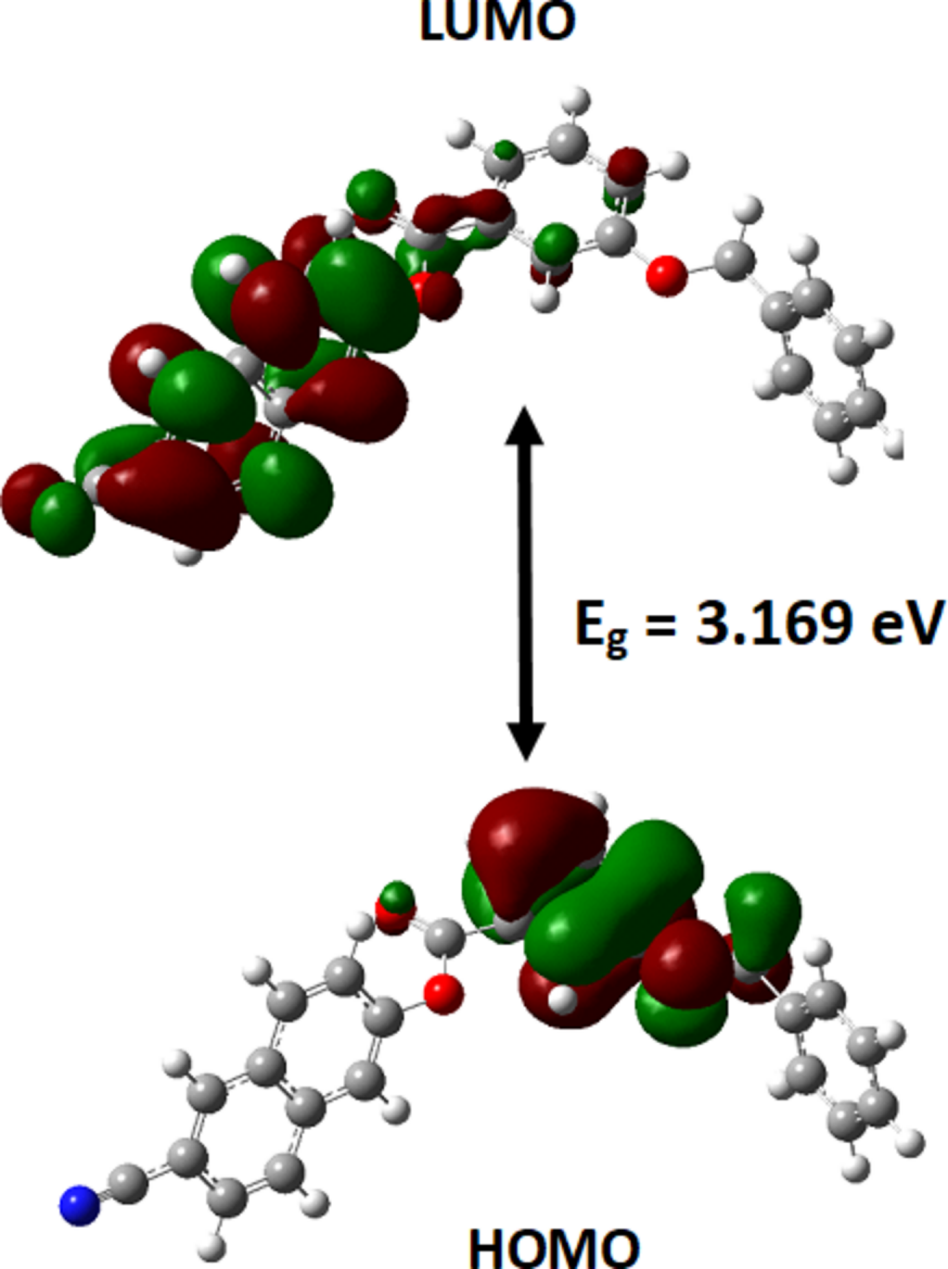
HOMO and LUMO of (I) with the energy band gap *E_g_*.

The energy absorbed between bonding (HOMO) and anti-bonding (LUMO) orbitals determines the band gap of the material. The HOMO and LUMO were generated and their energies evaluated from the optimized structure, as shown in Fig. 8 ▸. The electron density in the HOMO of the mol­ecule (I) mainly resides on the ester (O—C=O) group, and at the phenyl benzoate fragment to a lesser extent. In the LUMO, the electronic charge densities are delocalized to reside on the naphthalene ring and the ester group. The energies of HOMO and LUMO are −8.72 eV and −5.55 eV, respectively, resulting in an energy gap (*E_g_*) of 3.17 eV. Other parameters calculated in the DFT study are compiled in Table 2 ▸.

## 6. Mol­ecular Electrostatic Potential (MESP).

**Figure 9:**
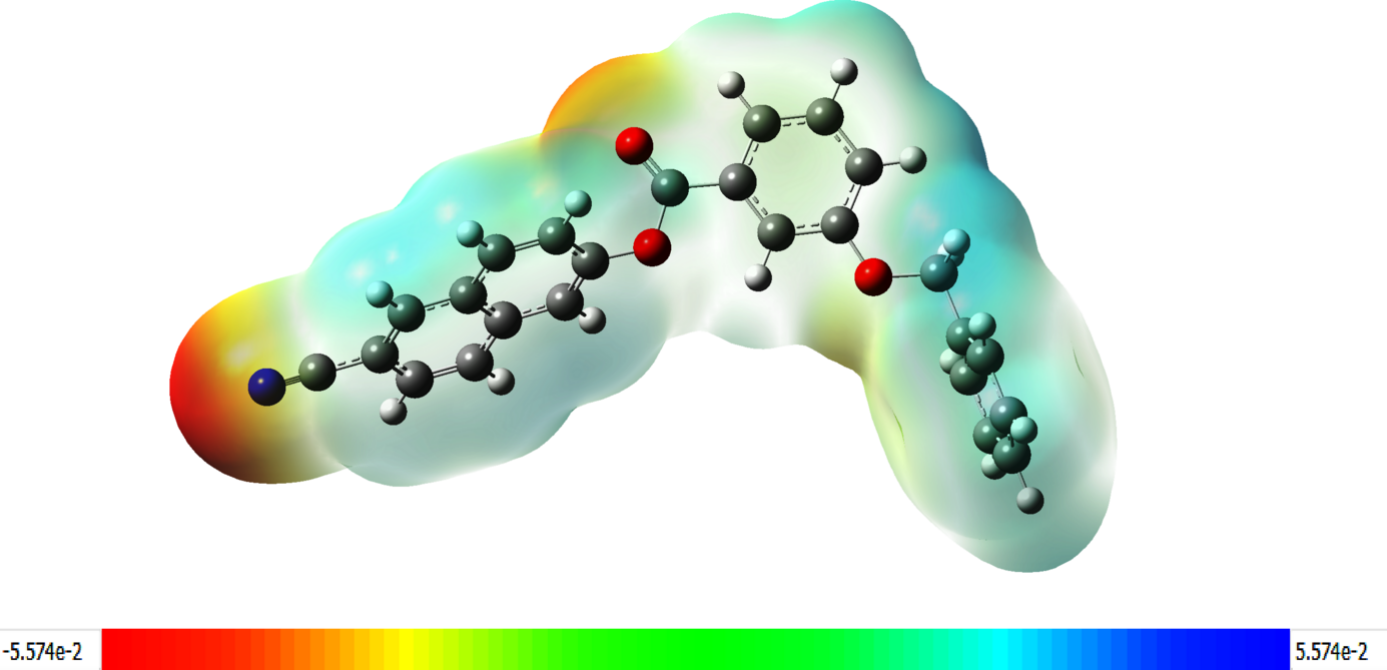
MEP plots of the title compound; regions of attractive potential appear in red and those of repulsive potential appear in blue.

The mol­ecular electrostatic potential surface (MEPS) can be used to visualize the electrostatic potential of a mol­ecule. For (I), the MEPS is illustrated in Fig. 9 ▸, which provides possible information about the reactive sites. The electron-rich part with a partial negative charge is shown by the combination of red and pale-yellow regions on the MEPS over the nitro­gen atom of the cyanona­phthalene moiety and the oxygen atom of the ester group, which is expected to undergo weak electrophilic attack. The faint blue colour spread all over the mol­ecule implies less electron deficient parts. The absence of a bright-blue region on the MEPS reveals that there are no possible sites on the mol­ecule for nucleophile attack (Friesner *et al.*, 2006 ▸).

## 7. Mol­ecular docking studies

**Figure 10:**
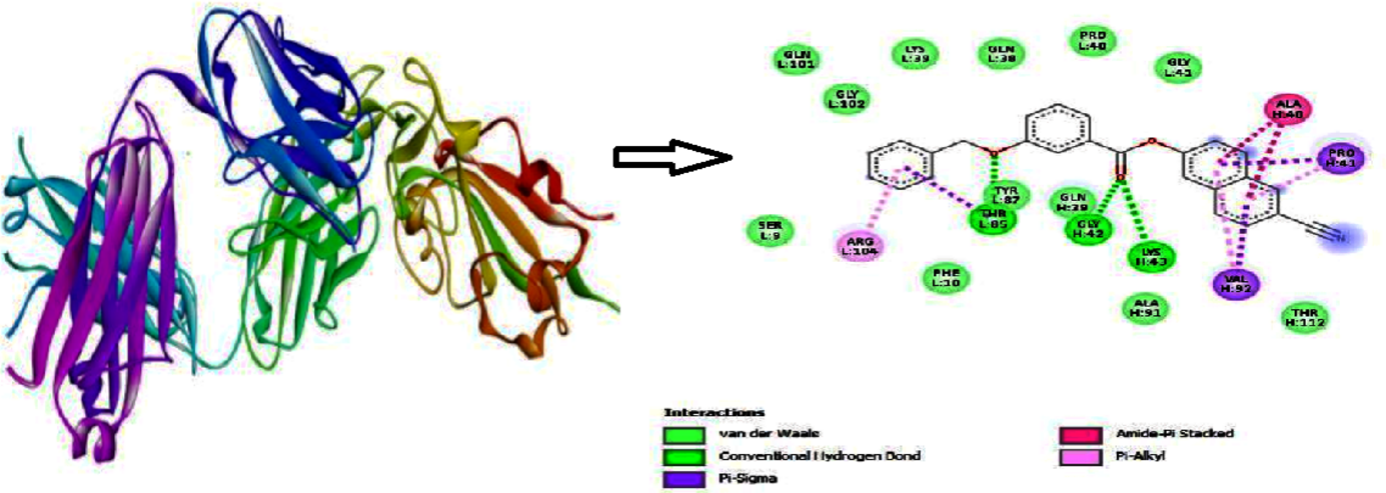
A three-dimensional view of the SARS-Covid-2(PDB ID:7QF0) protein and two-dimensional view of the mol­ecular inter­action between the ligand and amino acid residues.

*AutoDock Vina* (Morris *et al.*, 2009 ▸) was used to carry out the docking studies. The SARS-Covid-2(PDB ID:7QF0; Planchais *et al.*, 2022 ▸) protein was selected as a receptor and the title compound as a ligand. A good binding affinity score of −9.5 kcal mol^−1^ was obtained. The inter­action as generated by *Discovery Studio Visualizer* (Biovia, 2017 ▸) is shown in Fig. 10 ▸. It clearly illustrates that there are eleven hydrogen bonds and twelve van der Waals inter­actions between the ligand and the amino acid residues of the protein. Hence, the title mol­ecule can be considered as a potential candidate for pharmaceutical applications.

## 8. Database survey

A search in the Cambridge Crystallographic Database (CSD version 2.0.4 of December 2019; Groom *et al.*. 2016 ▸) for the mol­ecule containing a (benz­yloxy)benzoate fragment resulted in fourteen matches: in all these compounds, the torsion angles of the C—O—C—C unit indicate an *anti*-periplanar conformation. Among them, the compound with CCDC code VUCFEI (Harish Kumar *et al.*, 2024 ▸) is very similar to the title compound in which the cyano-biphenyl fragment is replaced by a cyano-naphthalene fragment. The search for mol­ecules containing cyanona­pthalene moieties resulted in twelve matches. In three of them, CIVZIR (Clegg *et al.*, 2008 ▸), IKUMOR (Li *et al.*, 2010 ▸) and KOPTIU (Baya *et al.*, 2015 ▸), a bulky group is attached to the cyanona­phthalene fragment, which widens the dihedral angle between the two aromatic rings of the naphthalene moiety to more than 2.57 (2)°. Otherwise the cyanona­phthalene fragment is nearly planar.

## 9. Synthesis and crystallization

6-Cyanona­phthalen-2-yl 4-(benz­yloxy)benzoate was synthesised by the Steglich esterification reaction method between 3-benzyl­oxybenzoic acid and 6-hy­droxy-2-naphtho­nitrile.

To a solution of 3-benzyl­oxybenzoic acid (0.228 g, 1.0 mol), 6-hy­droxy-2-naphtho­nitrile (0.169 g, 1.0 mol) and a catalytic amount of DMAP (0.05 g) in dry di­chloro­methane (25 ml), DCC (0.220 g, 1. 2 mol) was added in one portion and the reaction mixture was stirred in argon medium for 12 h. The precipitate was filtered off and the filtrate was evaporated. The crude product was purified by recrystallization from chloro­form, yield 65%; m.p. 396–398 K; IR: 3331, 2239, 1730, 1315, 1450, 1286, 1197, 1076, 1916, 740 cm^−1^; ^1^H NMR: 7.83 (*m*, 6H, Ar-H), 7.50 (*m*, 6H, Ar-H), 7.34 (*m*, 3H, Ar-4), 5.20 (*s*, 2H, –CH_2_O) ppm; ^13^C NMR: 169.2, 149.2, 135.7, 128.7, 123.8, 119.8, 115.6, 105.6, 71.2, 33.6, 26.124.4 ppm; elemental analysis: calculated C, 79.14; H, 4.52; N, 3.69%; found C, 79.19; H, 4.60; N, 3.75.

## 10. Refinement

Crystal data, data collection and structure refinement details are summarized in Table 3 ▸. H atoms were positioned geom­etrically (C—H = 0.93 Å) and refined as riding with *U*_iso_(H) = 1.2*U*_eq_(C).

## Supplementary Material

Crystal structure: contains datablock(s) I. DOI: 10.1107/S2056989024009964/wm5736sup1.cif

Structure factors: contains datablock(s) I. DOI: 10.1107/S2056989024009964/wm5736Isup2.hkl

Supporting information file. DOI: 10.1107/S2056989024009964/wm5736Isup3.cml

CCDC reference: 2391127

Additional supporting information:  crystallographic information; 3D view; checkCIF report

## Figures and Tables

**Table 1:** Hydrogen-bond geometry (Å, °) *Cg*2 and *Cg*4 are the centroids of the C3–C8 and C20–C25 rings, respectively.

*D*—H⋯*A*	*D*—H	H⋯*A*	*D*⋯*A*	*D*—H⋯*A*
C10—H10⋯O2^i^	0.93	2.68	3.579 (3)	164
C16—H16⋯*Cg*4^ii^	0.93	2.97	3.711 (3)	138
C23—H23⋯*Cg*4^iii^	0.93	2.76	3.611 (3)	153
C24—H24⋯*Cg*2^iv^	0.93	2.98	3.858 (3)	159

Symmetry codes: (i) 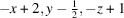; (ii) 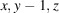; (iii) 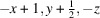; (iv) 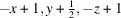.

**Table 2:** The energy values (eV) of global reactivity descriptors

*E*_HOMO	−8.72
*E*_LUMO	−5.55
Energy gap	3.17
Ionization energy	8.72
Electron affinity	5.55
Electronegativity	7.135
Electrophilicity index	16.059
Chemical hardness	1.585
Chemical softness	0.315 eV^−1^
Chemical potential	−7.135

**Table 3:** Experimental details

Crystal data	
Chemical formula	C_25_H_17_NO_3_
*M*_r_	379.39
Crystal system, space group	Monoclinic, *P*2_1_
Temperature (K)	285
*a*, *b*, *c* (Å)	9.3141 (3), 6.7593 (2), 15.3574 (5)
β (°)	105.163 (1)
*V* (Å^3^)	933.19 (5)
*Z*	2
Radiation type	Mo *K*α
μ (mm^−1^)	0.09
Crystal size (mm)	0.42 × 0.31 × 0.27

Data collection	
Diffractometer	Bruker SMART APEXII CCD
Absorption correction	Multi-scan (*SADABS*; Krause *et al.*, 2015 ▸)
*T*_min_, *T*_max_	0.966, 0.975
No. of measured, independent and observed [*I* > 2σ(*I*)] reflections	26288, 4790, 4314
*R*_int_	0.037
(sin θ/λ)_max_ (Å^−1^)	0.684

Refinement	
*R*[*F*^2^ > 2σ(*F*^2^)], *wR*(*F*^2^), *S*	0.049, 0.112, 1.11
No. of reflections	4790
No. of parameters	262
No. of restraints	1
H-atom treatment	H-atom parameters constrained
Δρ_max_, Δρ_min_ (e Å^−3^)	0.18, −0.26
Absolute structure	Flack *x* determined using 1567 quotients [(*I*^+^)−(*I*^−^)]/[(*I*^+^)+(*I*^−^)] (Parsons *et al.*, 2013 ▸)
Absolute structure parameter	0.0 (3)

Computer programs: *APEX2* and *SAINT* (Bruker, 2017 ▸), *SHELXT* (Sheldrick, 2015*a* ▸), *SHELXL* (Sheldrick, 2015*b* ▸), *Mercury* (Macrae *et al.*, 2020 ▸) and *publCIF* (Westrip, 2010 ▸).

## supplementary crystallographic information

### 6-Cyanonaphthalen-2-yl 4-(benzyloxy)benzoate Crystal data

**Table:**

C_25_H_17_NO_3_	*F*(000) = 396
*M**_r_* = 379.39	*D*_x_ = 1.350 Mg m^−^^3^
Monoclinic, *P*2_1_	Melting point: 386 K
Hall symbol: P 2yb	Mo *K*α radiation, λ = 0.71073 Å
*a* = 9.3141 (3) Å	Cell parameters from 2709 reflections
*b* = 6.7593 (2) Å	θ = 2.9–29.0°
*c* = 15.3574 (5) Å	µ = 0.09 mm^−^^1^
β = 105.163 (1)°	*T* = 285 K
*V* = 933.19 (5) Å^3^	Prism, colourless
*Z* = 2	0.42 × 0.31 × 0.27 mm

### 6-Cyanonaphthalen-2-yl 4-(benzyloxy)benzoate Data collection

**Table:**

Bruker SMART APEXII CCD diffractometer	4790 independent reflections
Radiation source: fine-focus sealed tube	4314 reflections with *I* > 2σ(*I*)
Graphite monochromator	*R*_int_ = 0.037
Detector resolution: 0.012 pixels mm^-1^	θ_max_ = 29.1°, θ_min_ = 2.9°
φ and Ω scans	*h* = −12→12
Absorption correction: multi-scan (*SADABS*; Krause *et al.*, 2015)	*k* = −8→9
*T*_min_ = 0.966, *T*_max_ = 0.975	*l* = −20→21
26288 measured reflections	

### 6-Cyanonaphthalen-2-yl 4-(benzyloxy)benzoate Refinement

**Table:**

Refinement on *F*^2^	Secondary atom site location: difference Fourier map
Least-squares matrix: full	Hydrogen site location: inferred from neighbouring sites
*R*[*F*^2^ > 2σ(*F*^2^)] = 0.049	H-atom parameters constrained
*wR*(*F*^2^) = 0.112	*w* = 1/[σ^2^(*F*_o_^2^) + (0.0503*P*)^2^ + 0.1406*P*] where *P* = (*F*_o_^2^ + 2*F*_c_^2^)/3
*S* = 1.11	(Δ/σ)_max_ < 0.001
4790 reflections	Δρ_max_ = 0.18 e Å^−^^3^
262 parameters	Δρ_min_ = −0.26 e Å^−^^3^
1 restraint	Absolute structure: Flack *x* determined using 1567 quotients [(*I*^+^)-(*I*^-^)]/[(*I*^+^)+(*I*^-^)] (Parsons *et al.*, 2013)
0.012 constraints	Absolute structure parameter: 0.0 (3)
Primary atom site location: structure-invariant direct methods	

### 6-Cyanonaphthalen-2-yl 4-(benzyloxy)benzoate Special details

**Table:**

Geometry. All esds (except the esd in the dihedral angle between two l.s. planes) are estimated using the full covariance matrix. The cell esds are taken into account individually in the estimation of esds in distances, angles and torsion angles; correlations between esds in cell parameters are only used when they are defined by crystal symmetry. An approximate (isotropic) treatment of cell esds is used for estimating esds involving l.s. planes.

### 6-Cyanonaphthalen-2-yl 4-(benzyloxy)benzoate Fractional atomic coordinates and isotropic or equivalent isotropic displacement parameters (Å^2^)

**Table:**

	*x*	*y*	*z*	*U*_iso_*/*U*_eq_	
O1	0.6200 (2)	0.4858 (3)	0.73167 (12)	0.0472 (5)	
O2	0.8441 (2)	0.3738 (3)	0.48087 (11)	0.0429 (4)	
O3	0.7449 (2)	0.0904 (3)	0.41447 (13)	0.0562 (5)	
N1	1.0966 (4)	0.8151 (5)	−0.00409 (19)	0.0720 (8)	
C20	0.5314 (3)	0.6692 (4)	0.84087 (16)	0.0404 (5)	
C25	0.4012 (3)	0.7744 (5)	0.82984 (18)	0.0484 (6)	
H25	0.314701	0.729814	0.789327	0.058*	
C24	0.3981 (3)	0.9466 (5)	0.8788 (2)	0.0545 (7)	
H24	0.309367	1.015313	0.871917	0.065*	
C23	0.5259 (4)	1.0150 (5)	0.93713 (17)	0.0519 (7)	
H23	0.524380	1.130395	0.969887	0.062*	
C22	0.6562 (3)	0.9121 (5)	0.94689 (19)	0.0559 (7)	
H22	0.743250	0.959386	0.985945	0.067*	
C21	0.6599 (3)	0.7402 (5)	0.8998 (2)	0.0510 (7)	
H21	0.748828	0.671363	0.907589	0.061*	
C19	0.5315 (3)	0.4734 (4)	0.7946 (2)	0.0522 (7)	
H19A	0.571340	0.372032	0.839136	0.063*	
H19B	0.430467	0.436973	0.763393	0.063*	
C15	0.6204 (3)	0.3220 (4)	0.67884 (16)	0.0369 (5)	
C14	0.6894 (3)	0.3444 (4)	0.60972 (16)	0.0362 (5)	
H14	0.733653	0.464269	0.602097	0.043*	
C13	0.6925 (2)	0.1874 (4)	0.55184 (15)	0.0348 (5)	
C18	0.6267 (3)	0.0076 (4)	0.56294 (16)	0.0413 (5)	
H18	0.627027	−0.096681	0.523488	0.050*	
C17	0.5613 (3)	−0.0141 (4)	0.63296 (18)	0.0473 (6)	
H17	0.519366	−0.135115	0.641378	0.057*	
C16	0.5568 (3)	0.1408 (4)	0.69109 (18)	0.0441 (6)	
H16	0.511664	0.124155	0.737942	0.053*	
C12	0.7611 (3)	0.2051 (4)	0.47543 (16)	0.0387 (5)	
C1	0.9033 (3)	0.4198 (4)	0.40816 (15)	0.0369 (5)	
C10	0.9968 (3)	0.2840 (4)	0.37968 (16)	0.0400 (5)	
H10	1.020892	0.163273	0.408940	0.048*	
C9	1.0511 (3)	0.3335 (4)	0.30832 (16)	0.0377 (5)	
H9	1.112080	0.244941	0.288451	0.045*	
C8	1.0162 (2)	0.5176 (4)	0.26402 (14)	0.0338 (5)	
C3	0.9284 (2)	0.6563 (3)	0.29738 (15)	0.0342 (5)	
C2	0.8722 (3)	0.6006 (4)	0.37101 (16)	0.0386 (5)	
H2	0.814018	0.688432	0.393638	0.046*	
C7	1.0641 (3)	0.5664 (4)	0.18674 (16)	0.0391 (5)	
H7	1.121836	0.477536	0.164345	0.047*	
C4	0.8978 (3)	0.8423 (4)	0.25446 (17)	0.0419 (5)	
H4	0.845363	0.936274	0.277969	0.050*	
C5	0.9433 (3)	0.8867 (4)	0.17954 (17)	0.0435 (6)	
H5	0.920636	1.008990	0.151554	0.052*	
C6	1.0253 (3)	0.7458 (4)	0.14457 (16)	0.0403 (5)	
C11	1.0668 (3)	0.7861 (4)	0.06199 (19)	0.0496 (6)	

### 6-Cyanonaphthalen-2-yl 4-(benzyloxy)benzoate Atomic displacement parameters (Å^2^)

**Table:**

	*U*^11^	*U*^22^	*U*^33^	*U*^12^	*U*^13^	*U*^23^
O1	0.0643 (11)	0.0366 (10)	0.0515 (10)	−0.0114 (8)	0.0341 (9)	−0.0087 (8)
O2	0.0574 (10)	0.0396 (10)	0.0374 (9)	−0.0067 (8)	0.0224 (8)	−0.0056 (7)
O3	0.0713 (13)	0.0535 (12)	0.0519 (11)	−0.0164 (10)	0.0305 (10)	−0.0191 (10)
N1	0.097 (2)	0.070 (2)	0.0591 (15)	0.0155 (17)	0.0381 (15)	0.0228 (14)
C20	0.0513 (14)	0.0390 (13)	0.0384 (12)	−0.0067 (11)	0.0251 (10)	−0.0015 (10)
C25	0.0464 (14)	0.0527 (17)	0.0452 (14)	−0.0026 (12)	0.0104 (11)	−0.0016 (12)
C24	0.0602 (17)	0.0483 (17)	0.0603 (17)	0.0132 (13)	0.0250 (14)	0.0054 (13)
C23	0.082 (2)	0.0397 (14)	0.0416 (13)	−0.0051 (14)	0.0290 (13)	−0.0055 (11)
C22	0.0584 (17)	0.0583 (18)	0.0483 (15)	−0.0152 (15)	0.0093 (13)	−0.0055 (13)
C21	0.0429 (13)	0.0525 (17)	0.0608 (16)	−0.0015 (12)	0.0193 (12)	−0.0012 (13)
C19	0.0694 (18)	0.0414 (16)	0.0587 (16)	−0.0104 (13)	0.0396 (14)	−0.0078 (12)
C15	0.0416 (12)	0.0332 (12)	0.0376 (11)	−0.0022 (9)	0.0135 (10)	−0.0029 (9)
C14	0.0411 (12)	0.0320 (12)	0.0370 (11)	−0.0026 (9)	0.0130 (9)	0.0010 (9)
C13	0.0360 (11)	0.0375 (13)	0.0310 (10)	0.0004 (9)	0.0086 (8)	0.0000 (9)
C18	0.0466 (13)	0.0359 (13)	0.0413 (12)	−0.0042 (11)	0.0113 (10)	−0.0070 (10)
C17	0.0545 (14)	0.0364 (14)	0.0532 (14)	−0.0126 (12)	0.0181 (12)	−0.0017 (11)
C16	0.0517 (14)	0.0433 (15)	0.0422 (13)	−0.0091 (11)	0.0210 (11)	−0.0009 (10)
C12	0.0416 (12)	0.0394 (13)	0.0355 (11)	−0.0010 (10)	0.0110 (9)	−0.0025 (10)
C1	0.0422 (12)	0.0380 (13)	0.0329 (11)	−0.0050 (10)	0.0138 (9)	−0.0054 (9)
C10	0.0470 (13)	0.0340 (12)	0.0395 (12)	0.0020 (10)	0.0123 (10)	0.0035 (10)
C9	0.0425 (12)	0.0342 (12)	0.0386 (11)	0.0037 (10)	0.0145 (10)	−0.0037 (9)
C8	0.0338 (10)	0.0347 (12)	0.0322 (10)	−0.0009 (9)	0.0076 (8)	−0.0033 (9)
C3	0.0372 (11)	0.0304 (11)	0.0339 (11)	0.0000 (9)	0.0072 (9)	−0.0063 (9)
C2	0.0436 (13)	0.0362 (13)	0.0379 (11)	0.0007 (10)	0.0140 (10)	−0.0079 (10)
C7	0.0423 (12)	0.0385 (13)	0.0382 (11)	0.0017 (10)	0.0132 (10)	−0.0032 (10)
C4	0.0504 (13)	0.0319 (13)	0.0446 (13)	0.0043 (10)	0.0143 (11)	−0.0043 (10)
C5	0.0542 (14)	0.0316 (13)	0.0423 (12)	0.0022 (11)	0.0082 (11)	0.0040 (10)
C6	0.0450 (13)	0.0411 (14)	0.0344 (11)	−0.0049 (11)	0.0096 (10)	0.0012 (10)
C11	0.0583 (16)	0.0443 (15)	0.0469 (14)	0.0027 (12)	0.0149 (12)	0.0083 (12)

### 6-Cyanonaphthalen-2-yl 4-(benzyloxy)benzoate Geometric parameters (Å, º)

**Table:**

O1—C15	1.373 (3)	C13—C12	1.481 (3)
O1—C19	1.427 (3)	C18—C17	1.375 (3)
O2—C12	1.368 (3)	C18—H18	0.9300
O2—C1	1.404 (3)	C17—C16	1.384 (4)
O3—C12	1.194 (3)	C17—H17	0.9300
N1—C11	1.137 (4)	C16—H16	0.9300
C20—C25	1.378 (4)	C1—C2	1.348 (4)
C20—C21	1.384 (4)	C1—C10	1.412 (3)
C20—C19	1.502 (4)	C10—C9	1.363 (3)
C25—C24	1.390 (4)	C10—H10	0.9300
C25—H25	0.9300	C9—C8	1.415 (3)
C24—C23	1.369 (4)	C9—H9	0.9300
C24—H24	0.9300	C8—C7	1.412 (3)
C23—C22	1.373 (4)	C8—C3	1.424 (3)
C23—H23	0.9300	C3—C4	1.413 (3)
C22—C21	1.374 (4)	C3—C2	1.416 (3)
C22—H22	0.9300	C2—H2	0.9300
C21—H21	0.9300	C7—C6	1.378 (4)
C19—H19A	0.9700	C7—H7	0.9300
C19—H19B	0.9700	C4—C5	1.360 (4)
C15—C14	1.386 (3)	C4—H4	0.9300
C15—C16	1.395 (4)	C5—C6	1.412 (4)
C14—C13	1.389 (3)	C5—H5	0.9300
C14—H14	0.9300	C6—C11	1.445 (4)
C13—C18	1.391 (4)		
			
C15—O1—C19	116.41 (19)	C17—C16—C15	119.4 (2)
C12—O2—C1	118.01 (18)	C17—C16—H16	120.3
C25—C20—C21	119.0 (3)	C15—C16—H16	120.3
C25—C20—C19	120.4 (3)	O3—C12—O2	122.9 (2)
C21—C20—C19	120.5 (3)	O3—C12—O2	122.9 (2)
C20—C25—C24	120.5 (3)	O3—C12—C13	125.2 (2)
C20—C25—H25	119.7	O2—C12—C13	111.91 (19)
C24—C25—H25	119.7	O2—C12—C13	111.91 (19)
C23—C24—C25	120.0 (3)	C2—C1—O2	116.9 (2)
C23—C24—H24	120.0	C2—C1—O2	116.9 (2)
C25—C24—H24	120.0	C2—C1—C10	122.7 (2)
C24—C23—C22	119.5 (3)	O2—C1—C10	120.3 (2)
C24—C23—H23	120.3	O2—C1—C10	120.3 (2)
C22—C23—H23	120.3	C9—C10—C1	118.5 (2)
C23—C22—C21	121.0 (3)	C9—C10—H10	120.7
C23—C22—H22	119.5	C1—C10—H10	120.7
C21—C22—H22	119.5	C10—C9—C8	121.1 (2)
C22—C21—C20	120.1 (3)	C10—C9—H9	119.5
C22—C21—H21	120.0	C8—C9—H9	119.5
C20—C21—H21	120.0	C7—C8—C9	121.7 (2)
O1—C19—C20	109.9 (2)	C7—C8—C3	119.1 (2)
O1—C19—H19A	109.7	C9—C8—C3	119.2 (2)
C20—C19—H19A	109.7	C4—C3—C2	122.5 (2)
O1—C19—H19B	109.7	C4—C3—C8	118.8 (2)
C20—C19—H19B	109.7	C2—C3—C8	118.6 (2)
H19A—C19—H19B	108.2	C1—C2—C3	119.7 (2)
O1—C15—C14	116.0 (2)	C1—C2—H2	120.1
O1—C15—C16	124.1 (2)	C3—C2—H2	120.1
C14—C15—C16	119.9 (2)	C6—C7—C8	119.9 (2)
C15—C14—C13	119.9 (2)	C6—C7—H7	120.0
C15—C14—H14	120.1	C8—C7—H7	120.0
C13—C14—H14	120.1	C5—C4—C3	121.4 (2)
C14—C13—C18	120.4 (2)	C5—C4—H4	119.3
C14—C13—C12	122.0 (2)	C3—C4—H4	119.3
C18—C13—C12	117.6 (2)	C4—C5—C6	119.5 (2)
C17—C18—C13	119.2 (2)	C4—C5—H5	120.3
C17—C18—H18	120.4	C6—C5—H5	120.3
C13—C18—H18	120.4	C7—C6—C5	121.1 (2)
C18—C17—C16	121.2 (2)	C7—C6—C11	118.8 (2)
C18—C17—H17	119.4	C5—C6—C11	120.1 (2)
C16—C17—H17	119.4	N1—C11—C6	178.3 (3)
			
C21—C20—C25—C24	−1.5 (4)	C14—C13—C12—O2	12.4 (3)
C19—C20—C25—C24	174.2 (2)	C18—C13—C12—O2	−169.2 (2)
C20—C25—C24—C23	1.3 (4)	O2—O2—C1—C2	0.0 (4)
C25—C24—C23—C22	−0.2 (4)	C12—O2—C1—C2	125.9 (2)
C24—C23—C22—C21	−0.8 (4)	C12—O2—C1—O2	0 (100)
C23—C22—C21—C20	0.6 (4)	O2—O2—C1—C10	0.0 (4)
C25—C20—C21—C22	0.5 (4)	C12—O2—C1—C10	−57.1 (3)
C19—C20—C21—C22	−175.1 (2)	C2—C1—C10—C9	−3.8 (4)
C15—O1—C19—C20	−174.8 (2)	O2—C1—C10—C9	179.5 (2)
C25—C20—C19—O1	116.3 (3)	O2—C1—C10—C9	179.5 (2)
C21—C20—C19—O1	−68.1 (3)	C1—C10—C9—C8	0.6 (4)
C19—O1—C15—C14	171.5 (2)	C10—C9—C8—C7	−176.2 (2)
C19—O1—C15—C16	−8.5 (4)	C10—C9—C8—C3	2.9 (3)
O1—C15—C14—C13	−178.7 (2)	C7—C8—C3—C4	−2.8 (3)
C16—C15—C14—C13	1.2 (3)	C9—C8—C3—C4	178.1 (2)
C15—C14—C13—C18	−0.1 (3)	C7—C8—C3—C2	175.6 (2)
C15—C14—C13—C12	178.3 (2)	C9—C8—C3—C2	−3.4 (3)
C14—C13—C18—C17	−1.3 (4)	O2—C1—C2—C3	−179.9 (2)
C12—C13—C18—C17	−179.7 (2)	O2—C1—C2—C3	−179.9 (2)
C13—C18—C17—C16	1.5 (4)	C10—C1—C2—C3	3.2 (4)
C18—C17—C16—C15	−0.4 (4)	C4—C3—C2—C1	178.9 (2)
O1—C15—C16—C17	178.9 (2)	C8—C3—C2—C1	0.5 (3)
C14—C15—C16—C17	−1.0 (4)	C9—C8—C7—C6	178.8 (2)
O2—O2—C12—O3	0.00 (4)	C3—C8—C7—C6	−0.3 (3)
C1—O2—C12—O3	4.7 (4)	C2—C3—C4—C5	−174.8 (2)
C1—O2—C12—O2	0 (100)	C8—C3—C4—C5	3.6 (3)
O2—O2—C12—C13	0.00 (7)	C3—C4—C5—C6	−1.2 (4)
C1—O2—C12—C13	−173.7 (2)	C8—C7—C6—C5	2.8 (4)
C14—C13—C12—O3	−166.0 (3)	C8—C7—C6—C11	−175.6 (2)
C18—C13—C12—O3	12.5 (4)	C4—C5—C6—C7	−2.1 (4)
C14—C13—C12—O2	12.4 (3)	C4—C5—C6—C11	176.3 (2)
C18—C13—C12—O2	−169.2 (2)		

### 6-Cyanonaphthalen-2-yl 4-(benzyloxy)benzoate Hydrogen-bond geometry (Å, º)

*Cg*2 and *Cg*4 are the centroids of the C3–C8 and C20–C25 rings, respectively.

**Table:**

*D*—H···*A*	*D*—H	H···*A*	*D*···*A*	*D*—H···*A*
C10—H10···O2^i^	0.93	2.68	3.579 (3)	164
C16—H16···*Cg*4^ii^	0.93	2.97	3.711 (3)	138
C23—H23···*Cg*4^iii^	0.93	2.76	3.611 (3)	153
C24—H24···*Cg*2^iv^	0.93	2.98	3.858 (3)	159

Symmetry codes: (i) −*x*+2, *y*−1/2, −*z*+1; (ii) *x*, *y*−1, *z*; (iii) −*x*+1, *y*+1/2, −*z*; (iv) −*x*+1, *y*+1/2, −*z*+1.
